# Anatomical Variants of Internal Carotid Artery—Results from a Retrospective Study

**DOI:** 10.3390/medicina59061057

**Published:** 2023-05-31

**Authors:** Bogdan Mihail Cobzeanu, Vasilica Baldea, Victor Vlad Costan, Mihail Dan Cobzeanu, Octavian Dragos Palade, Liliana Gheorghe, Luminita Radulescu, Florentina Severin, Corina Lupascu Ursulescu, Geanina Bandol, Cristian Martu, Andrei Mihail Rosu, Maria Luiza Cobzeanu

**Affiliations:** 1 Faculty of Medicine, University of Medicine and Pharmacy “Grigore T. Popa”, 700115 Iasi, Romania; bogdan-mihail.cobzeanu@umfiasi.ro (B.M.C.); eta.baldea@yahoo.com (V.B.); victor.costan@umfiasi.ro (V.V.C.); liliana.gheorghe@umfiasi.ro (L.G.); luminita.radulescu@umfiasi.ro (L.R.); florentina-s-severin@umfiasi.ro (F.S.); corina.ursulescu@umfiasi.ro (C.L.U.); geanina-i-afrasinei@d.umfiasi.ro (G.B.); martu.cristian@umfiasi.ro (C.M.); andrei-mihail-d-rosu@d.umfiasi.ro (A.M.R.); maria-luiza.cobzeanu@umfiasi.ro (M.L.C.); 2 Clinical Rehabilitation Hospital, 700661 Iasi, Romania; 3 Emergency Clinical Hospital “Sfântul Spiridon” Iasi, 700111 Iasi, Romania; 4 Regional Oncology Institute, 700483 Iasi, Romania

**Keywords:** internal carotid artery, anatomic variants, endoscopic sinus surgery, paranasal sinuses

## Abstract

*Background and Objectives*: The internal carotid artery (ICA) is a vascular structure that can be easily injured during sinus endoscopic procedures, and surgeons should be familiar with its anatomic variants. The aim of this study was to describe the anatomical variations in the internal carotid artery in relationship to sphenoidal sinuses, using computed tomography (CT). *Materials and Methods:* In this retrospective study, we evaluated the variations of the ICA in relationship to sphenoidal sinuses in a cohort of 600 patients who were assessed between January 2020 and December 2022 in ‘Saint Spiridon’ Emergency Hospital, Iasi, Romania. Descriptive statistics were used to characterize our data. *Results:* The most prevalent anatomical variant was represented by intrasinusal septa with posterior insertion on the ICA (58.6%), followed by procident ICA (58%) and dehiscent ICA (52%). We could not find any statistical significance regarding demographic characteristics among groups. *Conclusions:* A thorough CT examination should be performed before functional endoscopic sinus surgery, with the identification of anatomical variants of the ICA, in order to prevent its injury with potentially fatal consequences.

## 1. Introduction

One of the most commonly variable anatomical structures in the human anatomy is that of the nasal cavities and paranasal sinuses. Recognizing these anatomical elements is crucial for a sinus surgeon due to their intricate three-dimensional architecture and wide range of anatomical variances. One of the key strategies for treating chronic rhinosinusitis and a variety of other sinus illnesses is functional endoscopic sinus surgery (FESS) [1]. Prior to any surgical procedure, a complete evaluation needs to be conducted using computed tomography (CT) of the paranasal sinuses, which gives the anatomical “road map” to detect the existence of important structural anomalies with higher degree of clarity and precision [2].

The most common indications for FESS include chronic rhinosinusitis, acute rhinosinusitis (<4 weeks), and subacute rhinosinusitis (between 4 and 12 weeks) [3,4]. The contraindications of this procedure are represented by patients who have general contraindications for general or local anesthesia; lesions or disorders extending into the palate, skin or soft tissues, or laterally into or above the orbit; lateral recesses of the frontal sinus; or advanced intracranial involvement [3].

One of the most serious and dreaded side effects of endonasal transsphenoidal surgery is injury to the internal carotid artery (ICA), which has an estimated incidence of 0.1–0.3%, and may reach 5–9% in cases of prolonged endonasal approaches [2,5,6]. The incidence of this complication is much lower with improved surgical experience, CT scan quality, and higher resolution endoscopy.

The internal carotid artery possesses distinct anatomical characteristics in terms of its course and plays a vital role in providing blood supply to the brain. It exhibits variable curvatures along different segments of its pathway, underscoring its unique nature. Occasionally, the ICA may feature one or two flexures near the base of the skull. Furthermore, as it traverses the carotid canal and extends along the lateral aspect of the sphenoid bone, it assumes a serpentine or ‘S’-shaped configuration, resembling the shape of a cobra hood [7]. This intricate course highlights the complexity and significance of the ICA in cerebral circulation. Determining the relationships between the intra-cavernous and supra-cavernous ICA is crucial for the neurosurgeon when considering an endoscopic endonasal transsphenoidal approach.

In relation to the lateral wall of the sphenoid sinus, the internal carotid arteries can be situated at a distance from the sinus, within the sinus without indentation, or with varying degrees of procidentia, ranging from discrete forms to almost complete protrusion [8]. The bony lamella covering the carotid artery within the sphenoid sinus is an important element of interest in CT examination and can exhibit dehiscence.

There may be varying degrees of pneumatization in the sphenoid sinus and surrounding bony structures. Sphenoid sinuses are closely associated to several variants, including cavernous sinus, internal carotid artery, optic and vidian canals [9].

Multiple studies have demonstrated that the configuration of the internal carotid artery (ICA) and optic nerve can be influenced by the pneumatization of the sphenoid sinus, resulting in noticeable bulging on the sinus sidewall [8,10]. Furthermore, the sinus wall may exhibit regions where the artery and nerve are exposed without a protective bony covering. The involvement of the sphenoid sinus has been observed in approximately 65.2% of patients diagnosed with chronic sinusitis [11]. Additionally, the sphenoid sinus serves as a favorable pathway for accessing the anterior and middle cranial fossa during various surgical procedures, such as pituitary gland surgery.

The Onodi cell, also known as the sphenoethmoidal air cell, represents a distinct anatomical variation of the posterior ethmoid cell. This cell is characterized by its superior and lateral pneumatization towards the sphenoid sinus and its proximity to the optic nerve. A recent study by Jaworek-Troć et al. performed a retrospective analysis of 296 CT scans for the evaluation of the prevalence of the Onodi cell in the Polish population [12]. The authors reported a relatively frequent occurrence of the Onodi cell, which was observed in 31 patients, with a higher prevalence in males (20 patients) than females (11 patients). No lateral preference was observed for this anatomical pattern among the female cohort; on the other hand, the prevalence of the Onodi cell was observed to be higher on the left side in the male group. A bilateral presence of Onodi cells was observed in only one case.

In addition, a dehiscence in the bone that protects the internal carotid artery may cause the artery to come into touch with the sinus mucosa directly, which might result in an infection of the cavernous sinuses. On the other hand, carotid artery damage might cause blindness or a fatal hemorrhage if the surgeon was not aware of this variance prior to surgery [13].

During surgical procedures involving the carotid artery or optic nerve, surgeons must exercise utmost caution when encountering regions where these structures lack a protective bony covering. This is crucial to minimize the risk of inadvertent damage and ensure their preservation throughout the operation. According to the literature, complications of sinus surgery, such as orbital lesion, dural or intracranial injury, and damage to the internal carotid artery, range from 1.3 to 9.3% [14]. Intraoperative injury to the ICA during FESS is a rare but potentially life-threatening complication. The occurrence of such complications is infrequently reported in the literature, with only a limited number of documented case [9]. In cases of traumatic injury to the ICA during exploration of the sphenoid sinus, significant hemorrhage occurs, accompanied by severe limitations in accessing the sinus and impaired vision. Managing the profuse bleeding in such situations poses significant challenges, and, unfortunately, it can quickly become a life-threatening situation for patients, leading to fatal outcomes within minutes.

Limited data from clinical studies on various populations are available in the literature regarding the implications of anatomical variants of the ICA for FESS procedures. The aim of this study was to describe the anatomical variations of the internal carotid artery in relationship with sphenoidal sinuses using CT scans.

## 2. Materials and Methods

We performed an anatomic-radiological retrospective study that evaluated the variations of the ICA in relationship with sphenoidal sinuses in a cohort of 600 patients who were assessed between January 2020 and December 2022 in ‘Saint Spiridon’ Emergency Hospital from Iasi, Romania. Ethical approval for this study was obtained from the Institutional Ethics Committee of ‘Saint Spiridon’ Emergency Hospital (No. 44/02.05.2023).

Patients with various disorders (ophthalmological, neurological, dental, ear–nose–throat pathologies, or trauma) needing CT examinations were included in the study. The exclusion criteria comprised patients with age less than 18 years (such patients have incomplete anatomical structures), important artifacts on CT examinations revision surgery cases, or incomplete medical records.

The imaging protocol was performed using a Phillips AURA CT device and helicoidal technique on axial, coronal, and sagittal planes, with reconstruction of 1.0/1.0 mm to 1.0/1.0 mm slices (slice thickness/increment). Paranasal sinus CT images were obtained in axial projections. Coronal and sagittal images were reconstructed using the same (non-enhanced) bone shadow settings.

Based on the images acquired by the tomography scans, the following variables were assessed: the type of pneumatization of the sphenoidal sinus, the anatomic variations of the ICA, the presence of septations, and the position of the sphenoidal septum.

Medical records were also reviewed and demographic characteristics were retrieved: age, gender, medium of living. Descriptive statistics and univariate analysis of the variables were performed using chi-squared test and SPSS software version 25.0 (SPSS Inc., Chicago, IL, USA). A *p* values less than 0.05 was considered statistically significant.

## 3. Results

A total of 600 patients were included in this retrospective study. The first anatomical variant studied was ICAs at distance from sphenoidal sinus. In total, 150 cases were evaluated for the presence of this variant, and their description is presented in Table 1. The global prevalence of this anatomical variant in our cohort of patients was 54% (*n* = 81 patients). The majority of ICAs at distance from sphenoidal sinus (SS) were unilateral (55.5%, *n* = 45 patients), and in the left side (66.6%, *n* = 30 patients). We could not find any statistically significant difference between groups regarding their demographic characteristics.

**Figure 1:**
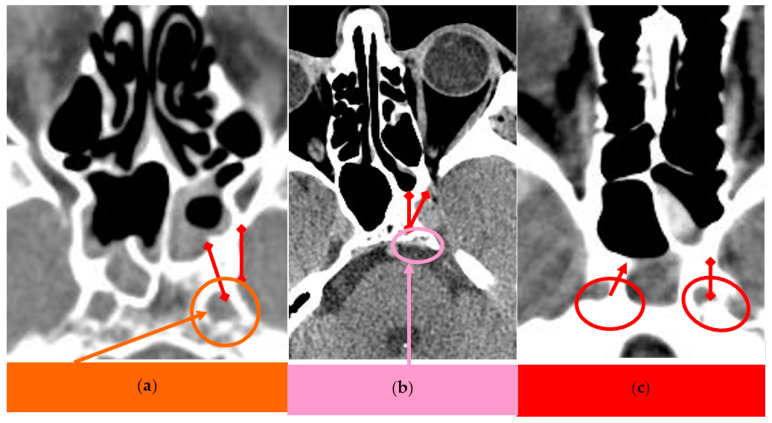
(**a**) Left ICA at distance from presellar SS. (**b**) Left ICA at distance from left conchal sinus. (**c**) ICAs at distance from sphenoidal sinuses.

Selected CT scans of the ICA at distance from SS sinus are presented in Figure 1a–c.

**Figure 2:**
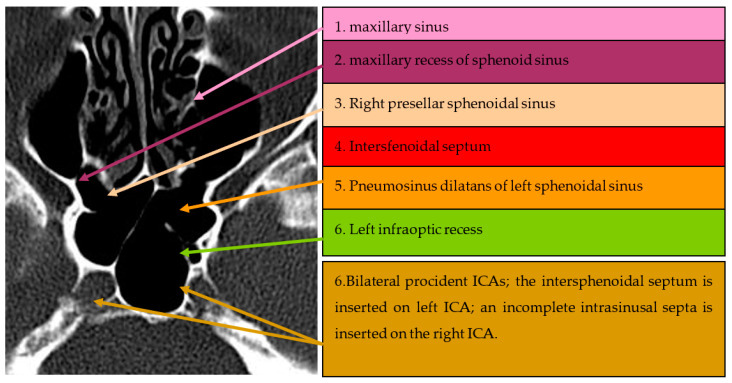
Axial CT section of paranasal sinuses with procidence of both ICAs, on which are inserted two septa.

The second anatomical variant studied was the procident ICA in 150 cases, presented in Table 2. The global prevalence of this anatomical variant in our cohort of patients was 58% (*n* = 87 patients). The majority of procident ICAs were bilateral (55.1%, *n* = 48 patients). We could not find any statistically significant difference between groups regarding their demographic characteristics, excepting the medium distribution of left and right procident ICAs (*p* = 0.016). A selected CT scan of the procident ICA is presented in Figure 2.

**Figure 3:**
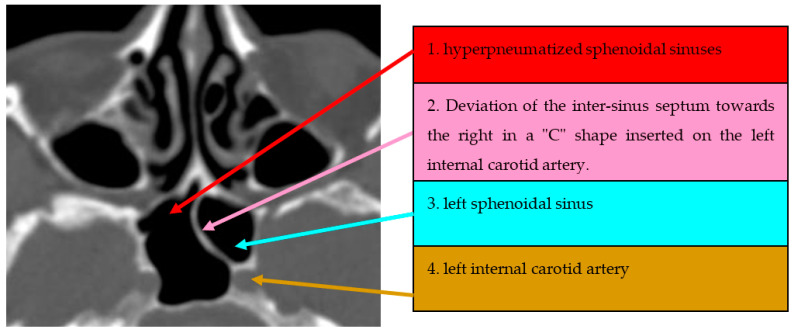
Axial CT section of paranasal sinuses with deviated intersphenoidal septum towards right, in a “C” shape, inserted on the internal carotid artery.

The third anatomical variant studied was the dehiscent ICA in 150 cases presented in Table 3. The global prevalence of this anatomical variant in our cohort of patients was 52% (*n* = 78 patients). The majority of dehiscent ICAs were unilateral (69.2%, *n* = 54 patients). We could not find any statistically significant difference between groups regarding their demographic characteristics. Selected CT scans of dehiscent ICAs are presented in Figure 3.

**Figure 4:**
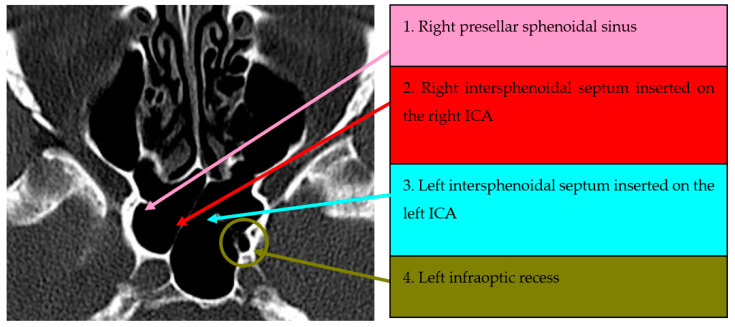
Axial CT section of paranasal sinuses. Right intersphenoidal septum. Incomplete left intrasphenoidal septum, posteriorly inserted on the ICA.

**Figure 5:**
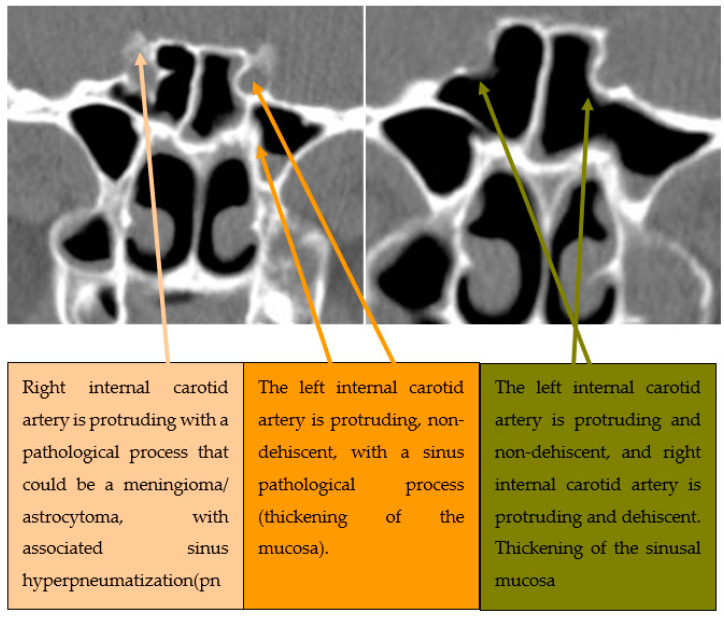
Coronal CT section of paranasal sinuses. Various degrees of protrusion of the internal carotid arteries.

The fourth anatomical variant studied was intrasinusal septa with posterior insertion on the ICA in 150 cases presented in Table 4. The global prevalence of this anatomical variant in our cohort of patients was 58.6% (*n* = 88 patients). The majority of intrasinusal septa were unilateral (75%, *n* = 66 patients), and on the left side (63.6%, *n* = 42 patients). We could not find any statistically significant difference between groups regarding their demographic characteristics. Selected CT scans of this anatomic variant are presented in Figure 4 and Figure 5.

## 4. Discussion

Endoscopic sinus surgery has emerged as the established and widely accepted approach for the treatment of chronic sinusitis. Acquiring a better understanding of the diverse regional anatomy of the sphenoid sinus plays a crucial role in reducing surgical complications associated with trans-sphenoidal and functional endoscopic sinus surgery. Previous studies have emphasized that the sphenoid sinus is recognized as the most variable cavity within the human body, posing challenges in terms of surgical access and intervention. By familiarizing themselves with the intricate anatomy of the sphenoid sinus, surgeons can enhance their surgical techniques, optimize patient outcomes, and minimize the risk of complications during these procedures.

This retrospective study provided an overview of the main anatomical variants of ICAs in relationship with the sphenoidal sinus. By definition, the internal carotid arteries, in their intracavernous course, exhibit a protrusion within the sphenoid sinus, similar to the optic nerve [15]. This results in a certain degree of indentation within the sinus. When this degree of indentation becomes abnormal, it manifests as the anatomical variation known as procidentia. This condition can be expressed in varying degrees, ranging from discrete forms to almost complete protrusion into the sinus, with or without dehiscence.

The most prevalent anatomical variant was represented by intrasinusal septa with posterior insertion on the ICA (58.6%), followed by procident ICA (58%) and dehiscent ICA (52%). We could not find any statistical significance regarding demographic characteristics among groups, and our results are in line with previously published literature [16,17].

Wide openings of the sphenoid sinus are often necessary for extended endoscopic endonasal approaches in order to enable simple access to the operated area and allow for adequate visualization of the posterior wall of the sinus. The septa from the sphenoid sinus must often be removed for this stage. A specific focus on the ICA anatomical course should be given during the opening of the sphenoid sinus. Internal carotid artery dehiscence and protrusion have a broad range of prevalence, ranging from 2% to 23% and 5.2% to 67%, respectively [18]. Rereddy et al. discovered a statistically significant correlation between dehiscent or protruding internal carotid artery and dehiscent or protruding optic nerve [19].

The optic nerve is located in the superolateral part of the sphenoid sinus and, similar to the internal carotid artery, its canal can exhibit dehiscence. Dehiscence of the optic nerve canal can also occur when it reaches the sphenoid or ethmoid sinus and leaves an osseous imprint on the lateral walls [18]. This serves as a significant surgical landmark when the optic nerve has an intrasinusal course. During endoscopic sinus surgery, an unintended fracture of the intersphenoidal septum that is connected to the bony wall of the ICA or optic nerve canal can lead to injury of these structures. This can result in significant intraoperative bleeding or even blindness. Therefore, utmost care must be taken to prevent such fractures and minimize the risk of complications during the surgical procedure.

The number of septa and the pattern of septation inside the sphenoid sinus seem to be unaffected by gender or ethnicity [20]. In total, 4.7% of cases have been documented to include carotid canal insertion of an intersphenoid sinus septum [21]. The sphenomaxillary plate is another anatomical variation in which the sphenoid sinus and the maxillary sinus are connected by a bony septum at the back. To prevent ocular damage during surgery, this structure has to be identified on the CT scans.

Examining the anatomical variations of the internal carotid artery (ICA) enables radiologists to provide more precise descriptions of patients who necessitate endoscopic endonasal approaches (EEA) to the skull base [22]. This comprehensive evaluation emphasizes key anatomical characteristics, including the classification and extensions of SS pneumatization, irregularities in the walls of the parasellar and paraclival segments of the ICA, the relative position of the septum with respect to the ICA, and the presence of septations within the SS.

Therefore, Fernandez-Miranda et al. [23] evaluated the anatomical relation between intrasphenoid septations and ICAs using 27 CT angiographic scans on live patients and 27 CT scans of fresh-frozen cadaveric heads. The authors reported 85% and 41% of the scans from the first group had at least one or two septa, respectively, touching one of the ICAs. When evaluating the second group, the authors concluded that 89% had at least one septation inserted in the ICAs.

The presence of sphenoidal septa that attach to the ICA has been identified as a potential risk factor for ICA injury during surgical procedures. Previous studies have established a correlation between septations within the sphenoid sinus and the ICA, with many intrasphenoidal septa inserting near the parasellar or paraclival carotid prominences [16,24]. Manipulation or dissection of these septa during surgery can result in damage to the ICA, as they are physically attached to the vessel wall and located in close proximity to these structures.

We only included adult patients, although patients older than 12 years old, when the sphenoid sinus achieves its full size and ultimate anatomical structure, may also have comparable findings. Pneumatization, which typically begins 12 months after birth, comes to a halt at this time [25]. During trans-sphenoidal pituitary surgery, postsellar pneumatization from the sphenoid sinus, especially pneumatization of the dorsum sella, may cause penetration of the posterior wall of the sphenoid and a cerebrospinal fluid (CSF) leak [26].

For the purposes of our study, we used CT axial scans, which were completely repetitive when compared to coronal scans and found to be superior in most studies for identifying septa in preoperative imaging, although one publication found that coronal scans were superior to axial scans for assessing septa [20,27].

A recent study by Dal Secchi et al., on 90 patients’ CT scans, revealed a prevalence of 26% for the protrusion of the parasellar ICA, and of 35% for the paraclival ICA. The same study indicated a prevalence of only 3.6% for the paraclival ICA [13]. Moreover, another study by Sasagawa et al., suggested that carotid artery protrusion and dehiscence occur more frequently among acromegalic patients [28]. It was also demonstrated that between 5 and 28% of patients with rhinosinusitis or facial trauma have internal carotid artery protrusion into the sphenoid sinus [29,30].

Both ICA procidence and dehiscence were included in the CT checklist before sellar and parasellar surgery, along with the presence of sphenoid sinus pneumatization or intersphenoid septa [30]. Unspecific symptoms may result from a dehiscent ICA canal, which is characterized by a thinning of the bony plate separating the ICA from the middle ear [31]. Prior to beginning surgical procedures, it is crucial to undergo the proper imaging to rule out potentially fatal complications as this illness sometimes resembles other middle-ear abnormalities [32].

The dehiscence of the osseous wall protecting the carotid canal is estimated at 6% in many studies [33]. This anatomical variation increases the risk of posttraumatic rupture or intraoperative injury during sphenoidectomy, leading to intrasinus hemorrhage through the branches of arteries that traverse the clivus. In some cases, pseudoaneurysm of the intracavernous internal carotid artery or dural fistula may develop [34]. Dehiscence, along with procidentia, further increases the risk of catastrophic hemorrhage, especially when combined with lateralization of the intersphenoidal septum or an intrasphenoidal septum attached to the vessel.

In addition, it is crucial to be well-prepared for other potential complications that may arise during FESS, including cerebrospinal fluid leak, retrobulbar hematoma, and bleeding from the internal carotid artery (ICA) [34]. Familiarity with the appropriate treatment modalities for each complication is essential. A laceration to the ICA during FESS procedure should be managed immediately with bilateral common carotid artery compression in the neck region; prompt compression of both common carotid arteries can significantly reduce bleeding (although it may not completely stop it due to collateral blood flow from the vertebral artery system), gauze packing, and even ligation of the artery [35]. Each surgeon performing FESS should be aware of the local anatomy and variants of important neurovascular structures, and must be able to promptly recognize intraoperative complications and their management.

This study has some limitations due to its retrospective, descriptive design on Caucasian patients. One strong point of this study is represented by a relatively large cohort of patients, with variate addressability to the imaging department in a 3-year time-frame. More powerful imaging techniques based on artificial intelligence could potentially determine with more accuracy the anatomical variants of ICA at the level of sphenoidal sinuses, and could offer surgeons a comprehensive anatomical picture before proceeding to FESS.

## 5. Conclusions

The preparedness of surgeons prior to conducting endoscopic skull base surgery through computed tomography scanning plays a crucial role in facilitating a favorable outcome. Specifically, it equips them with essential tools to anticipate the intraoperative findings, thereby mitigating the risk of iatrogenic complications such as inadvertent damage to the internal carotid artery or other delicate anatomical structures. Consequently, the utilization of preoperative CT imaging enables surgeons to effectively strategize and plan the surgical approach. It is imperative that radiologists accurately document the anatomical variations and their interrelationships for comprehensive reporting.

Although FESS is generally considered a safe procedure, the occurrence of carotid artery injury is a catastrophic complication that necessitates careful consideration during surgical planning. To mitigate this risk, it is crucial to identify patients with significant risk factors and conduct preoperative imaging to assess the anatomical variations and potential proximity of the carotid artery. Implementing a multidisciplinary approach and adhering to established management protocols are essential for minimizing morbidity and mortality associated with such complications.

## Figures and Tables

**Table 1:** Internal carotid artery at distance from sphenoidal sinus—case descriptions.

Demographic Characteristics	ICA at Distance from SS								
	Yes (*n* = 81)	No (*n* = 69)	*p* Value	Bilateral (*n* = 36)	Unilateral (*n* = 45)	*p* Value	Left (*n* = 30)	Right (*n* = 15)	*p* Value
Age, years (mean and standard deviation)	53.75 ± 18.87	53.20 ± 14.93	0.84	52.11 ± 18.79	55.07 ± 19.05	0.73	60.27 ± 15.87	64.67 ± 21.71	0.31
Gender (*n*/%)	Male = 34 (42%) Female = 47 (58%)	Male = 34 (49.3%) Female = 35 (50.7%)	0.37	Male = 16 (44.4%) Female = 20 (55.6%)	Male = 18 (40.0%) Female = 27 (60%)	0.61	Male = 13 (43.3%) Female = 17 (56.7%)	Male = 5 (33.3%) Female = 10 (66.7%)	0.56
Medium (*n*/%)	Urban = 36 (44.4%) Rural = 45 (55.6%)	Urban = 32 (46.4%) Rural = 37 (53.6%)	0.81	Urban = 17 (47.2%) Rural = 19 (52.8%)	Urban = 19 (42.2%) Rural = 26 (57.8%)	0.87	Urban = 10 (33.3%) Rural = 20 (66.7%)	Urban = 9 (60%) Rural = 6 (40%)	0.21

ICA—internal carotid artery; SS—sphenoidal sinus.

**Table 2:** Procident internal carotid artery—case descriptions.

Demographic Characteristics	Procident ICA								
	Yes (*n* = 87)	No (*n* = 63)	*p* Value	Bilateral (*n* = 48)	Unilateral (*n* = 42)	*p* Value	Left (*n* = 21)	Right (*n* = 21)	*p* Value
Age, years (mean and standard deviation)	60.90 ± 15.85	56.25 ± 17.43	0.10	62.46 ± 15.46	59.57 ± 16.08	0.13	60.27 ± 15.87	64.67 ± 21.71	0.31
Gender (*n*/%)	Male = 36 (41.4%) Female = 51 (58.6%)	Male = 30 (47.6%) Female = 33 (52.4%)	0.44	Male = 18 (37.5%) Female = 30 (62.5%)	Male = 20 (47.6%) Female = 22 (52.4%)	0.54	Male = 9 (42.9%) Female = 12 (57.1%)	Male = 11 (52.4%) Female = 10 (47.6%)	0.70
Medium (*n*/%)	Urban = 36 (41.4%) Rural = 51 (58.6%)	Urban = 28 (44.4%) Rural = 35 (55.6%)	0.70	Urban = 14 (29.2%) Rural = 34 (70.8%)	Urban = 23 (54.8%) Rural = 19 (45.2%)	0.05	Urban = 15 (71.4%) Rural = 6 (28.6%)	Urban = 8 (38.1%) Rural = 13 (61.9%)	0.016

ICA—internal carotid artery.

**Table 3:** Dehiscent internal carotid artery—case descriptions.

Demographic Characteristics	Dehiscent ICA								
	Yes (*n* = 78)	No (*n* = 72)	*p* Value	Bilateral (*n* = 24)	Unilateral (*n* = 54)	*p* Value	Left (*n* = 27)	Right (*n* = 27)	*p* Value
Age, years (mean and standard deviation)	54.83 ± 16.50	54.97 ± 14.77	0.95	50.79 ± 18.80	56.63 ± 15.22	0.31	53.11 ± 15.54	60.15 ± 14.31	0.15
Gender (*n*/%)	Male = 29 (37.2%) Female = 49 (62.8%)	Male = 28 (38.9%) Female = 44 (61.1%)	0.82	Male = 8 (33.3%) Female = 16 (66.7%)	Male = 21 (38.9%) Female = 33 (61.1%)	0.87	Male = 10 (37%) Female = 17 (63%)	Male = 11 (40.7%) Female = 16 (59.3%)	0.94
Medium (*n*/%)	Urban = 40 (51.3%) Rural = 38 (48.7%)	Urban = 33 (45.8%) Rural = 39 (54.2%)	0.50	Urban = 13 (54.2%) Rural = 11 (45.8%)	Urban = 27 (50%) Rural = 27 (50%)	0.75	Urban = 14 (51.9%) Rural = 13 (48.1%)	Urban = 13 (48.1%) Rural = 14 (51.9%)	0.93

ICA—internal carotid artery.

**Table 4:** Intrasinusal septa with posterior insertion on internal carotid artery—case descriptions.

Demographic Characteristics	Intrasinusal Septa with Posterior Insertion on Internal Carotid Artery								
	Yes (*n* = 88)	No (*n* = 62)	*p* Value	Bilateral (*n* = 27)	Unilateral (*n* = 66)	*p* Value	Left (*n* = 42)	Right (*n* = 24)	*p* Value
Age, years (mean and standard deviation)	57.09 ± 14.53	55.84 ± 15.33	0.12	55.85 ± 13.32	56.92 ± 14.98	0.08	53.11 ± 15.54	57.64 ± 14.23	0.19
Gender (*n*/%)	Male = 23 (26.1%) Female = 65 (73.89%)	Male = 19 (30.6%) Female = 43 (69.4%)	0.54	Male = 7 (25.9%) Female = 20 (74.1%)	Male = 18 (27.3%) Female = 48 (72.7%)	0.91	Male = 12 (28.6%) Female = 30 (71.4%)	Male = 6 (25%) Female = 18 (75%)	0.93
Medium (*n*/%)	Urban = 53 (60.2%) Rural = 35 (39.8%)	Urban = 34 (54.8%) Rural = 28 (45.2%)	0.51	Urban = 20 (74.1%) Rural = 7 (25.9%)	Urban = 38 (57.6%) Rural = 28 (42.4%)	0.13	Urban = 23 (54.8%) Rural = 19 (45.2%)	Urban = 15 (62.5%) Rural = 9 (37.5%)	0.82

## Data Availability

Data available to the corresponding author due to local policies.
